# Kolb’s Learning Style Inventory 4.0 and its association with traditional and problem based learning teaching methodologies in medical students

**DOI:** 10.12669/pjms.37.1.2275

**Published:** 2021

**Authors:** Muhammad Zafar Iqbal Hydrie, Syed Muhammad Zulfiqar Hyder Naqvi, Shams Nadeem Alam, Syed Imtiaz Ahmed Jafry

**Affiliations:** 1 Dr. Muhammad Zafar Iqbal Hydrie, MBBS, MPhil, PhD, Postdoc Department of Community Medicine, ***Present Address:*** Professor, School of Public Health, Dow University of Health Sciences, Ojha Campus, Karachi. Baqai Medical University, Karachi, Pakistan; 2 Dr. Syed Muhammad Zulfiqar Hyder Naqvi, MSBE. Department of Community Medicine, Baqai Medical University, Karachi, Pakistan; 3 Dr. Shams Nadeem Alam, FRCS, MHPE. Department of Medical Education, Baqai Medical University, Karachi, Pakistan; 4 Dr. Syed Imtiaz Ahmed Jafry, MPH. Department of Community Medicine, Baqai Medical University, Karachi, Pakistan

**Keywords:** Academic success, Problem-Based Learning, Students, medical, Teaching methods

## Abstract

**Objectives:**

To assess learning styles and the association of various teaching methodologies of medical students.

**Methods:**

A cross-sectional study was carried out amongst 523 medical students of Baqai Medical College, Baqai Medical University, Karachi, from July 2019 to October 2019. All students from first to final year, who attended the undergraduate MBBS program were included. The study instrument was a questionnaire containing students’ demographic details, David Kolb’s Learning Style Inventory 4.0 and traditional and PBL teaching methodologies were asked. The association of various learning styles and preferred teaching methodologies with year of study was also assessed by using Pearson’s chi-square test.

**Results:**

Out of 523 students, 518 returned the completed questionnaire. A majority of the students had either imagining or experiencing learning style. No change in learning style was observed between years of study. A significant association between the teaching methodologies and year of study was found in the imagining (p=0.033) and experiencing (p=0.044) learning style groups.

**Conclusion:**

Students from different years of study at medical school did not have significantly different learning styles though the student’s preferences to teaching methodologies seem to change over time in the respective learning style groups. Longitudinal studies are necessary to identify the factors influencing such change and explore the association between learning styles over time on teaching methodologies in medical education.

## INTRODUCTION

Kolb’s (1984) experiential learning cycle remains one of the most widely influential and cited model of experiential learning theory with emphasis on specific place and time.^1^ Pipitone and Raghavan highlighted the importance of social interactions, engagement with local community and intentional cultural narrative activities in explaining the learning experience.^2^ Previous research using Kolb’s Learning Style Inventory (KLSI) version 3.1 which identified four learning styles— Diverging, Assimilating, Converging, and Accommodating and showed that learning styles are influenced by culture, personality type, educational specialization, career choice, and current job role and tasks.^1^,^3^ Every learning style has got its own suitable instructional strategies and studies have shown a link between the two.^4^

Over the years many researchers observed that the four Kolb’s learning style types had a number of borderline cases which caused confusion due to overlapping of the learning styles. These original four learning styles have recently been refined into a nine style typology that better defines the unique patterns of individual learning styles and helps reduce the confusion introduced by borderline cases in the old 4 style typology.^3^,^5^ The new nine styles defined in KLSI version 4.0 are Initiating, Experiencing, Imagining, Reflecting, Analyzing, Thinking, Deciding, Acting and Balancing.^3^

Learning is an exercise which demands students to act pragmatically to find solutions, through an inquiry process and clear understanding of their purposeful roles and responsibilities.^6^ Thus learning can be problem-based or project-based process utilizing learning methodologies such as terms associated with experiential learning which include inquiry-based learning, student-directed learning, active learning, problem-based learning, service learning, and project-based learning etc.^7^ There is an emphasis on learner’s choice empowering them to make decisions and giving autonomy.^8^ Learning styles and instructional strategies in a society could be affected by many variables and understanding these helps in selecting the instructional strategies best suited to learners in the society.^9^ Thus the educator or teacher plays a very important role in facilitating the process, such as assisting learners to remain open to trying novel solutions to problems, encouraging tenacious attitudes, and promoting the effectiveness of communication skills.^10^ The learning process is often progressively difficult and educators need to gradually increase the difficulty of the intellectual, social, emotional, and/or physical challenge as the academic process proceeds ahead as seen with increasing years of study.^11^

Knowing the distribution of learning styles amongst medical students is therefore necessary to define which teaching methodologies will be most suited to these students. Some of the teaching methodologies such as interactive lectures, small group discussions and self-study were found to be associated with specific learning styles as reported by Costa et al. in their study.^12^ Similar observations were reported locally by Mukhtar et al. which found that the students preferred interactive lectures.^13^

In the given context, the aim of this study was to identify the various learning styles of undergraduate medical students by using the newly defined KLSI 4.0 and to determine their association with preferred teaching methodologies.

## METHODS

A cross-sectional study was carried out among the medical sciences students of Baqai Medical University, Karachi, Pakistan, from July 2019 to October 2019. Ethical approval (Ref: No: FHM 275-2019, Dated July 2, 2019) for the study was taken from Baqai Institute of Health Sciences.

Taking the percentage frequency of the study outcome as 50% for the most liberal estimate, with 95% confidence level and 5% precision, the minimum required sample size was calculated to be 385 participants. A total of 523 students belonging to medical, dental, pharmacy and physiotherapy institutes of Baqai Medical University were approached using convenience sampling technique. The filling of the questionnaire by the students was considered as their consent for participation in the study. Students belonging to all educational years, i.e. from first to final year were included in the study, but since BDS and DPT only have four year course durations, and many of the final year medical students were busy in their clinical postings and could not be easily reached in the university campus, the proportion of fifth year students in the final sample was quite low.

An anonymous study questionnaire including KLSI versions 3.1 was administered to 523 undergraduates first to final year students present on the campus. The independent variables of the study were gender, year of study, and preferred teaching methodologies while learning styles were the dependent variable of the study.

The Kolbs Learning Style Inventory (KLSI) 4.0 was used to assess learning styles, it has 20 items in this format—12 that are similar to the items in the KLSI 3.1, which has been previously well validated in medical students, and eight additional items that are about learning in different contexts.^3^ The second part of the questionnaire was to identify their preferences for teaching methodologies which were broadly divided into traditional and problem based learning (PBL).

The data were analyzed using Statistical Package for Social Sciences SPSS (version 23.0). Descriptive analysis was performed by calculating mean and standard deviation for age and frequency and percentages for gender, learning styles, and teaching methodologies. Inferential analysis was performed using chi-square test while the significance level was set at 0.05.

## RESULTS

A total of 518 students completed the forms that were included in the final analysis with a response rate of 99.0%. The mean age of the medical students was 21.5±1.69 years while 307 (59.3%) of them were females. Using David Kolb’s LSI 4.0 nine style typology, it was found that 271 (52.3%) of the medical students had Imagining, 181 (34.9%) had Experiencing, 35 (6.8%) had Reflecting while 25 (4.8%) had Balancing learning style. These four learning styles out of the nine style typology accounted for 98.8% of the learning styles in this study. Moreover, 3 (0.6%) students had Initiating, 2 (0.4%) had Thinking while 1 (0.2%) had Acting learning style, accounting for a total of 1.2% of the learning styles. Deciding and Analyzing learning styles were not found in any medical student in this study (Table-I).

**Table 1:** Students’ Kolb’s LSI 4.0 Nine Learning Styles (n=518*).

Learning Style	Count (%)
Imagining	271 (52.3)
Experiencing	181 (34.9)
Reflecting	35 (6.8)
Balancing	25 (4.8)
Initiating	3 (0.6)
Thinking	2 (0.4)
Acting	1 (0.2)

* There were no students in the Deciding and Analyzing categories.

A majority (n=452, 87.2%) of the students had Imagining or Experiencing learning styles in this study. Comparing the Kolbs 4.0 with Kolbs 3.1 learning styles we observed that of the total students with Imagining learning style (n=271), a majority were Divergers (n=205, 75.6%) while the remaining were Assimilators (n=66, 24.4%). The Experiencing learning style (n=181, 34.9%) included all four learning styles of Kolb`s learning style 3.1 in the order of Accommodators (n=83, 45.9%), Divergers (n=63, 34.8%), Convergers (n=25, 13.8%) and lastly Assimilators (n=10, 5.5%). It was observed that the distribution of students in the learning style groups did not change significantly with their year of study. The students in our study group predominantly adopted the Imagining and Experiencing learning styles in all years with 79.7% (n=55) in first year, 81.4% (n=70) in second year, 90.7% (n=156) in third year, 88.8% (n=150) in fourth year and 95.5% (n=21) in fifth year.

The teaching methods were grouped mostly as hybrid (both traditional and PBL methods) while traditional and PBL were also identified separately by the university students. Many students did not specify any teaching methods and they were grouped together as non-specific to show no preference to any teaching method. The study results showed a significant association between teaching methodologies and year of study (p=0.006) with most first, second, third and final year students preferring hybrid teaching method while most fourth year students preferring non-specific teaching method. Table-II

**Table-II:** Preferred teaching methodologies according to years of study (n=518).

Teaching Methods	1^st^ Year (n=71)	2^nd^ Year (n=88)	3^rd^ Year (n=173)	4^th^ Year (n=169)	5^th^ Year (n=22)

	Frequency (%)	Frequency (%)	Frequency (%)	Frequency (%)	Frequency (%)

Hybrid	32 (45.1)	33 (37.5)	57 (32.9)	52 (30.8)	13 (59.1)
Traditional	7 (9.9)	10 (11.4)	16 (9.2)	18 ( 10.7)	1 (4.5)
Problem based Learning	16 (22.5)	17 (19.3)	48 (27.7)	22 (13.0)	3 (13.6)
Non-Specific	16 (22.5)	28 (31.8)	52 (30.1)	77 (45.6)	5 (22.7)
p	0.004				

In this study, students having learning styles according to KLSI 4.0 were also seen for association with teaching methodology as year of study changes. It was observed that those with imagining (n=271) and experiencing (n=181) learning styles had significant association with year of study (p=0.033 and p=0.044 respectively). The other teaching methodologies were not found to have any association with year of study, probably due to small sample sizes in their respective categories. Table-III

**Table-III:** Kolb’s 4.0 learning styles with teaching methods and year of study (n=518).

*Learning Style*	*1^st^ Year (n=69 )*	*2^nd^ Year (n=86 )*	*3^rd^ Year (n=172)*	*4^th^ Year (n=169)*	*5^th^ Year (n=22)*	*p*

	Frequency (%)	Frequency (%)	Frequency (%)	Frequency (%)	Frequency (%)	

Imagining (n=271)	34 (49.3%)	42 (48.8%)	85 (49.4%)	99 (58.6%)	11 (50.0%)	0.033
Hybrid teaching (n=96)	16 (16.7%)	15 (15.6%)	25 (26%)	32 (33.3%)	8 (8.3%)	
PBL teaching (n=58)	8 (13.8%)	5 (8.6%)	27 (46.6%)	18 (31%)	Nil	
Traditional (n=24)	3 (12.5%)	4 (16.7%)	4 (16.7%)	12 (50%)	1 (4.2%)	
Non-specific (n=93)	7 (7.5%)	18 (19.4%)	29 (31.2%)	37 (39.8%)	2 (2.2%)	
Experiencing (n=181)	21 (30.4%)	28 (32.6%)	71 (41.3%)	51 (30.2%)	10 (45.5%)	0.044
Hybrid teaching (n=68)	9 (13.2%)	9 (13.2%)	29 (42.6%)	16 (23.5%)	5 (7.4%)	
PBL teaching (n=31)	4 (12.9%)	6 (19.4%)	15 (48.4%)	4 (12.9%)	2 (6.5%)	
Traditional (n=23)	3 (13%)	6 (26.1%)	11 (47.8%)	3 (13%)	Nil	
Non-specific (n=59)	5 (8.5%)	7 (11.9%)	16 (27.1%)	28 (47.5%)	3 (5.1%)	
Reflecting (n=35)	8 (11.6%)	9 (10.5%)	6 (3.5%)	12 (7.1%)	Nil	0.300
Hybrid teaching (n=11)	4 (36.4%)	3 (27.3%)	1 (9.1%)	3 (27.3%)	Nil	
PBL teaching (n=7)	2 (28.6%)	3 (42.9%)	2 (28.6%)	Nil	Nil	
Traditional (n=2)	Nil	Nil	Nil	2 (100%)	Nil	
Non-specific (n=15)	2 (13.3%)	3 (20%)	3 (20%)	7 (46.7%)	Nil	
Balancing (n=25)	5 (7.2%)	4 (4.7%)	9 (5.2%)	6 (3.6%)	1 (4.5%)	0.156
Hybrid teaching (n=6)	1 (16.7%)	3 (50%)	2 (33.3%)	Nil	Nil	
PBL teaching (n=7)	2 (28.6%)	1 (14.3%)	3 (42.9%)	Nil	1 (14.3%)	
Traditional (n=3)	1 (33.3%)	Nil	1 (33.3%)	1 (33.3%)	Nil	
Non-specific (n=9)	1 (11.1%)	Nil	3 (33.3%)	5 (55.6%)	Nil	

## DISCUSSION

Social scientists acknowledge the increasing role of medical education in understanding students’ learning styles and their role in achieving academic success.^14^ To improve academic success in students and increase their motivation to learn, it is important to identify their learning styles and to regulate the educational programs accordingly. The goals of medical schools can be better achieved by encouraging students to identify their own learning styles and realize their strong and weak points in learning, instructing them about how to improve their weaknsses, and raising awareness of the students’ learning styles among tutors and teachers.^15^,^16^ In our study, we defined learning styles using the revised David Kolb’s 4.0 nine style typology to evaluate the learning styles among the students of a medical university. More than half of the students were found to have Imagining learning style which according to the previous Kolb’s 3.1 version included Divergers and Assimilators. As we could not find any study using the new KLSI 4.0 so we compared studies which used KLSI 3.1 and found that most studies done in Pakistan and surrounding countries such as Turkey and Saudi Arabia have reported Divergers as predominant style, as also seen in our study using KLSI 4.0, which is the major of the two groups included in Imagining learning style in the new nine style typology.^17^,^18^ According to KLSI 3.1, the other group in the Imagining KLSI 4.0 is Assimilators and a previous study has found medical students to predominantly have assimilating learning style.^14^ It was determined in another study using KLSI 3.1 that the greatest change between the learning styles took place in the group of Divergers where a great majority of these students shifted to the assimilating learning styles over time.^19^,^20^ Divergers and Assimilators are the two learning styles according to KLSI 3.1 present in the new 4.0 version of Imagining learning style we could not detect any change if students shifted from Diverger to Assimilator learning style or vice versa in the new typology. The next predominant learning style in our study according to KLSI 4.0 was Experiencing learning style (n=181, 34.9%) and included all the learning styles of KLSI 3.1 in the order of mostly accommodators followed by Divergers, Convergers and a few Assimilators.

In our study students were also asked which teaching methodology was preferred by them. The results in a study done using KLSI 3.1 by Gurpinar et al., aimed at determining which educational methods are commonly preferred by medical students and increase their success the majority showed that assimilators were more successful in the courses based on traditional education while Convergers were more successful in the courses based on PBL.^18^ In our study, the overall major preference was for a combined teaching methodology since medical students adopt a variety of teaching methods to boost their learning potential depending on the course requirements and nature of study. The result of the present study also showed that medical students had a change in preference to the teaching methods over the year of study. Nearly 90% of the medical students in this study had either Imagining or Experiencing learning styles and both learning styles were found to be significantly associated with the year of study.

### Limitations of the study

As this was a cross-sectional study, we could only compare the students in different years of study and not follow them prospectively. A longitudinal cohort study is needed to be planned to continue to follow and observe the same students over an extended period of time. Results from such a cohort will help to determine whether medical education influences learning styles and vice versa, which incites their change as suggested by our study.

## CONCLUSION

Different teaching methods at the medical college did not significantly influence the students’ learning styles, however students preferred teaching methods and individual learning styles were affected by their year of study. Studies planned for a longer duration following students as they progress through their pre-clinical and clinical careers are needed to further explore the factors which may influence medical students preferred teaching methods and learning strategies.

### Authors’ Contribution:

**MZIH** conceived, designed and did data collection, statistical analysis & manuscript writing.

**SMZHN & SNA** did review & editing of manuscript.

**SIAJ** did final approval of manuscript.

**MZIH, SMZHN, SNA & SIAJ** ensured accuracy and integrity of work.
